# Burden of respiratory syncytial virus-associated lower respiratory infections in children in Spain from 2012 to 2018

**DOI:** 10.1186/s12879-022-07261-1

**Published:** 2022-03-31

**Authors:** Marco Heppe-Montero, Stefan Walter, Valentín Hernández-Barrera, Ruth Gil-Prieto, Ángel Gil-de-Miguel

**Affiliations:** 1 Department of Preventive Medicine and Public Health, Rey Juan Carlos University, Alcorcón, Madrid, Spain; 2 Hospital Infantil Universitario Niño Jesús, Madrid, Spain

**Keywords:** Respiratory syncytial virus, Bronchiolitis, Bronchitis, Pneumonia, Disease burden, Hospitalization, Epidemiology, Children, Spain

## Abstract

**Background:**

Respiratory syncytial virus (RSV) is a major cause of acute lower respiratory tract infection (ALRI) leading to infant hospitalization, morbidity and postnatal mortality in children younger than 5 years of age worldwide. The aim of this study was to collect data on hospitalizations for RSV-related ALRI in children in Spain from 2012 to 2018.

**Methods:**

We used the discharge reports from the Minimum Basic Data Set (MBDS) to retrospectively analyze hospital discharge data in children ≤ 14 years of age with a diagnosis of acute lower respiratory tract infection, based on the ICD-9-CM and ICD-10-CM diagnosis codes, from 2012 to 2018.

**Results:**

A total of 190,474 children, 58.1% boys and 41.9% girls, were admitted for lower respiratory tract infections in Spain, including 118,731 cases of bronchiolitis, 53,972 cases of bronchitis, 3710 cases of RSV-positive pneumonia, and 14,061 cases of RSV infections. Of these, 92,426 children (48.5%) had laboratory-confirmed RSV infection. The mean case fatality rate was almost 6 times higher for pneumonia (0.6%) than for bronchiolitis (0.1%) or bronchitis (0.1%). A significant linear increase in the mean annual hospitalization rate for pneumonia of almost 15% per year was found, with no changes in the trend over the study period.

**Conclusions:**

RSV-related respiratory infections remain a leading cause of infant hospitalization in Spain. Effective antiviral treatments and preventive vaccines are urgently needed for the management of RSV infection in children, especially for those aged 6 to 12 months.

## Background

Respiratory syncytial virus (RSV) is a major cause of acute lower respiratory tract infection (ALRI) leading to infant hospitalization, morbidity and postnatal mortality in children younger than 5 years of age worldwide, especially in developing countries. Data suggest that most RSV-related childhood mortality occurs during the first year of life [1–9]. Hospitalizations associated with RSV infection are highly seasonal, with annual epidemics generally occurring during the cold season in temperate climates [7, 10]. Although RSV infections occur frequently in all age groups, peak rates occur in infants aged 6 weeks to 6 months, especially in those younger than 3 months [3, 11]. It is believed that by 2 years of age, 90% of children have had RSV infection at least once, and half of them even twice [7, 12–14]. Roughly, one to two out of every 100 children younger than 6 months of age with RSV infection may need hospitalization [15] and, according to data from the US, up to 22% of children under 24 months of age may require admission to the intensive care unit (ICU) during their hospital stay [16]. These data suggest that the risk of infant mortality due to RSV infection in settings with inadequate intensive care facilities could increase alarmingly, even in children without previous underlying conditions.

The global health and economic burden of RSV-associated disease is considerable and poses a major global health problem. However, clinical data for global RSV-related mortality are scarce. Results from a recent extensive systematic review of both published studies and unpublished data estimated that in 2015, up to 33.1 million episodes of RSV ALRI occurred globally in children younger than 5 years, resulting in about 3.2 million hospital admissions and 59,600 (48,000–74,500) in hospital deaths, 99% of which occurred in developing countries and almost half (45%) (27,300 deaths, 20,700–36,200) among children under 6 months of age [17]. Data from the RSV GOLD study, a retrospective analysis of individual data from children under 5 years of age who died with RSV between January 1, 1995 and October 31, 2015 in hospitals across the world, showed a striking median age difference for RSV-related deaths between low/lower-to-middle-income countries and high-income countries of 5 and 84 months, respectively. According to the authors, young age at death in low-income and middle-income countries might reflect limitations in healthcare quality or access to care, rather than specific susceptibility of the children [18].

In Spain, results from an observational retrospective survey that evaluated all hospitalizations related to RSV infection in children up to 5 years of age between 1997 and 2011 in almost all hospitals in the country (98%) showed that the highest incidence of hospitalization occurred during the first year of life, 4137 per 100,000 children (95% CI 4121–4153), and up to 80% of deaths were registered in this population. The average annual cost to the Spanish national health system for bronchiolitis requiring hospitalization was 47 million euros, with an average cost per hospitalization of 2162 euros in children up to 5 years old [19]. Another study that analyzed the hospital discharge reports of children under 5 years of age with RSV infection in a reference pediatric hospital in northern Spain from 2010 to 2015 found that most children (72%) were younger than 1 year of age and 95% were previously healthy infants. Of all children younger than 5 years, 6.5% required direct admission or intermediate transfer to the pediatric ICU during their hospital stay. The percentage of infants under 2 months of age was higher than in children not admitted to the pediatric ICU (52% vs. 26%, *p* = 0.003) and the comorbidity rate was also higher (14% vs. 5%, *p* = 0.009). The case fatality rate among children admitted for RSV was 0.3% [20].

The most common clinical manifestation of ALRI in RSV-infected infants is bronchiolitis, which may lead to complications such as acute respiratory failure and apneas, but pneumonia and croup are also seen [7, 21, 22]. Prematurity, young age, chronic lung disease, congenital heart disease, immunosuppression, and neuromuscular disorders are risk factors for severe outcomes in RSV infection [15, 23]. Although the case fatality rate is highest in children with underlying conditions, most cases of life-threatening RSV infection occur among previously healthy children.

Natural RSV infection does not confer long-lasting immunity against subsequent infection and no specific treatment or widely available prevention options for RSV infections are available to date. Thus, treatment for RSV disease in patients with severe ALRI consists primarily of supportive care, supplemental oxygen, and mechanical ventilation if needed [24, 25]. Ribavirin, a synthetic guanosine analogue and broad-spectrum antiviral agent, is the only antiviral agent available for the treatment of life-threatening RSV infections. However, its use is currently limited to immunocompromised patients because of efficacy, safety, and cost issues, and the need for hospital admission for prolonged aerosol administration [26, 27]. RSV immunoprophylaxis (IP) is highly effective in preventing severe RSV infections in high-risk infants and young children. Palivizumab (Synagis), a recombinant humanized monoclonal IgG antibody, is currently the only market-approved IP for the prevention of RSV ALRI requiring hospitalization in children at high risk for RSV disease [26, 28, 29]. Recently, based on the positive data from the first interim analysis of the Phase 1 study NCT04528719, the US Food and Drug Administration (FDA) granted fast-track designation to Moderna’s investigational single-dose vaccine against RSV mRNA-1345 for use in adults older than 60 years of age [30]. Several RSV vaccines and monoclonal antibodies and direct-acting antiviral agents are currently in clinical development for the prevention and treatment, respectively, of RSV disease [25].

Accurate assessment of the burden of RSV hospitalization in children, together with identification of the population at greatest risk and the seasonal patterns of the virus, is essential to determine priorities for the use of existing therapeutic and prophylactic treatments against RSV in the context of limited healthcare resources, and to make the case for the rational development of novel, effective vaccines and treatments against RSV.

In this population-based study, we aimed to collect data on the burden of hospital admissions caused by RSV-associated bronchitis, bronchiolitis, and pneumonia in children up to 14 years of age in Spain between 2012 and 2018.

## Methods

### Study design and data sources

The purpose of this study was to collect data on hospitalizations for RSV-related ALRI in children in Spain from 2012 to 2018. We used discharge reports from the Minimum Basic Data Set (MBDS) published annually by the Spanish Ministry of Health to retrospectively analyze hospital discharge data containing a diagnosis of RSV infection, bronchitis, bronchiolitis, or pneumonia for the general Spanish population over a 7-year period from 2012 to 2018. The MBDS reports more than 90% of admissions to both public and private acute care hospitals in Spain and is validated for data quality and overall methodology by the Spanish Ministry of Health [31, 32].

For each MBDS discharge record, we retrieved up to 7 diagnoses coded on the basis of the International Classification of Diseases (ICD), Ninth Revision, Clinical Modification, ICD-9-CM, for cases collected from 2012 to 2015, and ICD-10-CM for cases collected from 2016 to 2018. All hospital admissions coding for RSV infection, bronchitis, bronchiolitis, or pneumonia in any diagnostic position in children aged up to 14 years of age throughout the period 2012–2018 were recorded. For each record, the following variables were collected: sex, diagnosis, and outcome (discharge/death). Eligible respiratory system diseases are listed in Table 1.

**Table 1:** Definition of diseases of the respiratory system used according to the ICD-9-CM and ICD-10-CM codes

ICD-9-CM codes (cases collected from 2012 to 2015)	
079.6	Respiratory syncytial virus
466.0	Acute bronchitis
466.1	Acute bronchiolitis
466.11	Acute bronchiolitis due to respiratory syncytial virus
480.1	Pneumonia due to respiratory syncytial virus
ICD-10-CM codes (cases collected from 2016 to 2018)	
B97.4	Respiratory syncytial virus as the cause of diseases classified elsewhere
J12.1	Respiratory syncytial virus pneumonia
J20.5	Acute bronchitis due to respiratory syncytial virus
J20.9	Acute bronchitis, unspecified
J21.0	Acute bronchiolitis due to respiratory syncytial virus
J21.9	Acute bronchiolitis, unspecified

### Data analysis

The number of hospitalizations examined per year was expressed as absolute frequencies (n). The hospitalization rate (by year and sex) per 100,000 population, in-hospital fatality rate (by year), and the ratio between confirmed deaths and confirmed cases (%) were calculated with 95% confidence intervals (CI). Hospitalization rates and case fatality rates were calculated separately for cases coded as RSV-positive respiratory disease and those coded as unspecified respiratory disease. Corrected population data from municipal records, extracted from the National Institute of Statistics [33], were used as the denominator for the hospitalization rates.

Hospitalization rates were compared using the unpaired Student’s t test to assess differences between sex groups, and analysis of variance (ANOVA) was used to assess differences between the study years and the diagnostic codes. Joinpoint regression models were used to evaluate trends in hospital admissions for RSV-related diseases over the study period [34]. This method provides the annual percentage change (APC) in hospitalization rates between the trend change points and estimates the average APC (AAPC) over the entire study period. When there are no joinpoints (i.e., no changes in the trend), APC is constant and equals AAPC. In all tests, the level of significance was set at *p* < 0.05. For the statistical analysis, we used IBM SPSS Statistics for Windows, Version 19.0 (Armonk, NY: IBM Corp), Stata Statistical Software, Release 11 (College Station, TX: StataCorp LP) and Joinpoint Software, Version 4.8.0.1 (National Cancer Institute, National Institutes of Health, Bethesda, MD, USA). The personal information of each subject was delivered to the researchers anonymously, in strict compliance with the current Spanish and European legislation. The project received a waiver from the local ethics committee, Comité de Ética de la Investigación de la Universidad Rey Juan Carlos, which ruled that no formal ethics approval was required.

## Results

A total of 190,474 children up to 14 years of age were hospitalized in Spain due to RSV-related diseases from 2012 to 2018. Of these, 58.1% were boys and 41.9% girls. The median age was 2 months (interquartile range [IQR] 4) for children under 1 year of age and 2 years (IQR 2) for those older than 1 year. Overall, of the three main RSV-related diseases addressed in our study, the most prevalent diagnosis was ‘bronchiolitis’ (ICD-9-CM 466.1 and 466.11; ICD-10-CM J21.0 and J21.9), that accounted for 62.3% of all hospitalizations (68,899 cases in boys [36.2%] and 49,832 cases in girls [26.2%]), followed by ‘bronchitis’ (ICD-9-CM 466.0; ICD-10-CM J20.5 and J20.9), 28.3% (31,870 cases in boys [16.7%] and 22,102 cases in girls [11.6%]), ‘RSV infection’ (ICD-9-CM 079.6; ICD-10-CM B97.4), 7.4% (8009 cases in boys [4.2%] and 6052 cases in girls [3.2%]), and ‘pneumonia’ (ICD-9-CM 480.1; ICD-10-CM J12.1), 1.9% (1919 cases in boys [1.0%] and 1791 cases in girls [0.9%]). Among children hospitalized for bronchiolitis (118,731 cases), 36.5% (25,974 cases in boys [59.8%] and 17,421 cases in girls [40.2%]) were diagnosed with ‘acute bronchiolitis’ (ICD 9 CM 466.1; ICD 10 CM J21.9) and 63.5% (42,925 cases in boys [57.0%] and 32,411 cases in girls [43.0%]) with ‘bronchiolitis due to RSV’ (ICD 9 CM 466.11; ICD 10 CM J21.0), while among those hospitalized for bronchitis (53,972 cases), 94.4% (30,120 cases in boys [59.1%] and 20,823 cases in girls [40.9%]) were diagnosed with ‘acute bronchitis’ (ICD 9 CM 466.0; ICD 10 CM J20.9) and 5.6% (1750 cases in boys [57.8%] and 1279 cases in girls [42.2%]) with ‘bronchitis due to RSV’ (ICD 10 CM J20.5). Almost half of all children, 92,426 (48.5%), had laboratory-confirmed RSV infection.

**Fig. 1:**
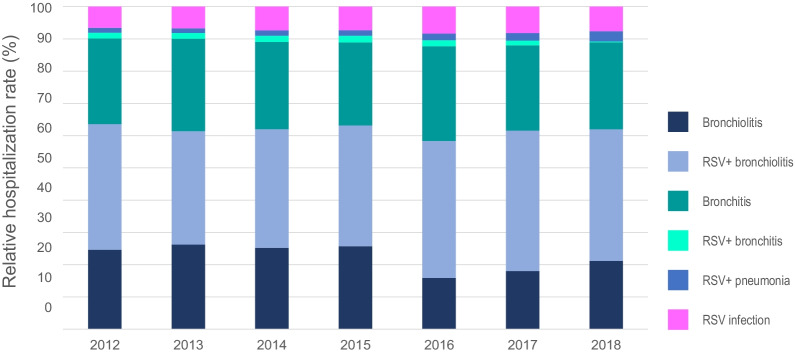
Distribution of the relative hospitalization rates (%) for bronchiolitis, RSV-positive bronchiolitis, bronchitis, RSV-positive bronchitis, RSV-positive pneumonia, and RSV infection by year in children up to 14 years of age in Spain, from 2012 to 2018

**Fig. 2:**
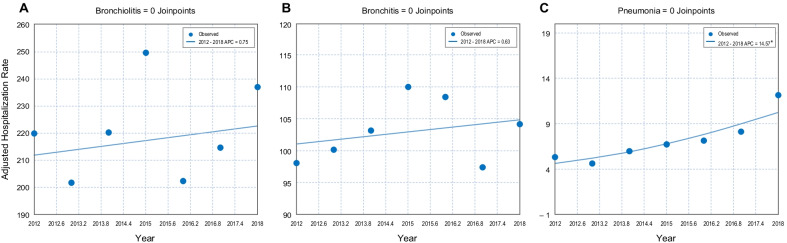
Joinpoint regression analysis of the adjusted hospitalization rates for bronchiolitis (**A**), bronchitis (**B**), and pneumonia (**C**) by year, from 2012 to 2018. Asterisk indicates that the annual percentage change (APC) is significantly different from zero at the alpha = 0.05 level

The mean hospitalization rate (per 100,000 population) during the study period was 26.7 (95% CI 25.4–27.9) for ‘RSV infection’ (29.4 [27.7–31.2] in boys and 23.7 [22.2–25.4] in girls), ranging from 22.1 (95% CI 21.1–23.2) in 2013 to 29.2 (95% CI 27.9–30.4) in 2018. For bronchiolitis diagnosis codes, the mean hospitalization rate (per 100,000 population) was 80.3 (95% CI 78.2–82.4) for ‘acute bronchiolitis’ (93.0 [90.1–96.1] in boys and 66.8 [64.2–69.4] in girls), ranging from 55.2 (95% CI 53.5–56.9) in 2016 to 101.7 (95% CI 99.5–104.0) in 2015, and 140.7 (95% CI 137.7–143.6) for ‘bronchiolitis due to RSV’ (155.3 [151.5–159.3] in boys and 125.1 [121.5–128.7] in girls), ranging from 115.5 (95% CI 113.2–117. 9) in 2013 to 156.0 (95% CI 153.1–159.00) in 2018 (Tables 2, 3 and Fig. 1). Using joinpoint regression analysis, no evidence of statistically significant changes in the mean annual hospitalization rate due to bronchiolitis or changes in the trend (APC = 0.75) was found throughout the period analyzed (Fig. 2A). For bronchitis diagnosis codes, the mean hospitalization rate (per 100,000 population) was 97.4 (95% CI 95.0–99.8) for ‘acute bronchitis’ (111.4 [108.1–114.8] in boys and 82.5 [79.5–85.5] in girls), ranging from 91.9 (95% CI 89.8–94.0) in 2012 to 103.1 (95% CI 100.8–105.6) in 2018, and 5.7 (95% CI 5.4–6.0) for ‘bronchitis due to RSV’ (6.4 [5.6–7.2] in boys and 5.0 [4.3–5.8] in girls), ranging from 1.1 (95% CI 0.8–1.3) in 2018 to 8.3 (95% CI 7.6–9.0) in 2015 (Tables 2, 3 and Fig. 1). Using joinpoint regression analysis, no evidence of statistically significant changes in the mean annual hospitalization rate due to bronchiolitis or changes in the trend was found throughout the period analyzed (APC = 0.63) (Fig. 2B). Finally, the mean hospitalization rate for ‘pneumonia due to RSV’ was 7.2 (95% CI 6.3–8.0) per 100,000 population (7.1 [6.3–8.0] in boys and 6.2 [5.5–7.1] in girls), ranging from 4.6 (95% CI 4.2–5.1) in 2013 to 12.2 (95% CI 11.4–13.0) in 2018 (Tables 2, 3 and Fig. 1). Joinpoint regression analysis did show a statistically significant increase in the mean annual hospitalization rate for pneumonia of 14.6% per year (*p* < 0.05), with no changes in the trend over the study period (Fig. 2C). Hospitalization rates (per 100,000 children) for both bronchiolitis and bronchitis were higher in boys than in girls: 248.4 vs. 191.8 and 117.8 vs. 87.4, respectively (*p* < 0.001). No significant sex differences were found for pneumonia (Table 3).

**Table 2:** Hospitalization rates due to RSV-related diseases by year in children in Spain from 2012 to 2018

Year	Bronchiolitis		RSV + bronchiolitis		Bronchitis		RSV + bronchitis		RSV + pneumonia		RSV infection	
	N	Annual rate^a^	N	Annual rate	N	Annual rate	N	Annual rate	N	Annual rate	N	Annual rate
2012	7190	85.24 (83.26–87.26)	11,384	134.91 (132.43–135.84)	7372	91.86 (89.78–93.98)	510	6.21 (5.68–6.77)	435	5.28 (4.78–5.80)	1865	22.7 (21.68–23.76)
2013	6835	86.31 (84.28–88.39)	9147	115.51 (113.15–117.89)	7319	94.29 (92.16–96.49)	463	5.9 (5.35–6.45)	364	4.63 (4.18–5.14)	1736	22.11 (21.10–23.20)
2014	7114	89.76 (87.67–91.90)	10,361	130.73 (128.23–133.16)	7311	96.3 (94.12–98.55)	528	6.86 (6.31–7.48)	459	5.96 (5.40–6.55)	2018	26.1 (24.98–27.27)
2015	7912	101.7 (99.46–103.97)	11,524	148.07 (145.37–150.83)	7541	101.73 (99.46–104.08)	618	8.27 (7.62–8.95)	503	6.72 (6.12–7.35)	2185	28.93 (27.72–30.16)
2016	4158	55.21 (53.52–56.93)	11,078	147.41 (144.66–147.66)	7574	102.01 (99.71–104.35)	474	6.43 (5.88–7.01)	523	7.13 (6.55–7.77)	2165	28.98 (27.76–30.23)
2017	4565	62.85 (61.04–64.71)	11,018	152.02 (149.17–153.33)	6672	92.37 (90.18–94.60)	363	5.05 (4.55–5.58)	582	8.13 (7.48–8.80)	2070	28.6 (27.38–29.87)
2018	5621	81.02 (78.91–83.17)	10,824	156.02 (153.07–159.00)	7154	103.12 (100.76–105.53)	73	1.05 (0.80–1.34)	844	12.17 (11.37–13.01)	2022	29.15 (27.91–30.43)
Total	43,395	80.3 (78.17–82.43)	75,336	140.67 (137.70–143.63)	50,943	97.38 (95.00–99.77)	3029	5.68 (5.41–5.95)	3710	7.15 (6.33–7.97)	14,061	26.65 (25.39–27.91)

^a^Annual hospitalization rate expressed per 100,000 population (95% CI)

**Table 3:** Hospitalization rates due to RSV-related diseases by sex in children in Spain from 2012 to 2018

	Boys			Girls			Total		
	N	%	Annual rate^a^	N	%	Annual rate	N	%	Annual rate
Bronchiolitis	25,974	59.9%	93.0 (90.1–96.1)	17,421	40.2%	66.8 (64.2–69.4)	43,395	100.0%	80.3 (78.3–82.3)
RSV + bronchiolitis	42,925	57.0%	155.3 (151.5–159.3)	32,411	43.0%	125.1 (121.5–128.7)	75,336	100.0%	140.7 (138.0–142.6)
Bronchitis	30,120	59.1%	111.4 (108.1–114.8)	20,823	40.9%	82.5 (79.5–85.5)	50,943	100.0%	97.4 (95.2–99.7)
RSV + bronchitis	1750	57.8%	6.3 (5.6–7.2)	1279	42.2%	5.0 (4.3–5.8)	3029	100.0%	5.7 (5.2–6.2)
RSV + pneumonia	1919	51.7%	7.1 (6.3–8.0)	1791	48.3%	6.2 (5.5–7.1)	3710	100.0%	7.1 (6.6–7.8)
RSV infection	8009	57.0%	29.4 (27.7–31.2)	6052	43.0%	23.7 (22.2–25.4)	14,061	100.0%	26.7 (25.5–27.8)

^a^Annual hospitalization rates expressed per 100,000 population (95% CI)

By age group, the mean annual hospitalization rates were highest in children under 12 months of age, especially those for bronchiolitis diagnosis codes, 1296.2 (95% CI 1283.1–1309.3) for ‘acute bronchiolitis’ and 2185.2 (95% CI 2168.2–2202.2) for ‘bronchiolitis due to RSV’, rates that were more than 10 times higher than those in children aged one year. In general, an age-related decrease in hospitalization rates was observed, especially in children ≤ 5 years of age, being more pronounced among children diagnosed with bronchiolitis (both ‘acute bronchiolitis’ and ‘bronchiolitis due to RSV’), followed by ‘acute bronchitis’ and ‘RSV infection’, while hospitalization rates due to ‘bronchitis due to RSV’ and ‘pneumonia due to RSV’ remained relatively low in all age groups (Table 4).

**Table 4:** Hospitalization rates due to RSV-related diseases by age group in children in Spain from 2012 to 2018

Age (years)	Bronchiolitis^a^	RSV + bronchiolitis	Bronchitis	RSV + bronchitis	RSV + pneumonia	RSV infection
< 1	1296.2 (1283.1–1309.3)	2185.2 (2168.2–2202.2)	722.0 (712.2–731.8)	45.3 (42.9–47.7)	52.1 (49.5–54.7)	292.7 (286.5–298.9)
1	113.27 (109.5–117.04)	197.95 (192.97–202.93)	328.89 (322.47–335.31)	26.03 (24.22–27.84)	34.91 (32.82–37)	84.75 (81.49–88.01)
2	32.64 (30.65–34.63)	80.13 (77.01–83.25)	220.97 (215.78–226.16)	15.29 (13.93–16.65)	19.86 (18.3–21.42)	45.77 (43.41–48.13)
3	15 (13.67–16.33)	36.82 (34.73–38.91)	134.66 (130.66–138.66)	6.36 (5.49–7.23)	8.12 (7.14–9.1)	18.61 (17.12–20.1)
4	7.35 (6.43–8.27)	19.25 (17.76–20.74)	78.67 (75.65–81.69)	1.99 (1.51–2.47)	2.98 (2.39–3.57)	9.16 (8.13–10.19)
5	4.49 (3.78–5.2)	11.49 (10.35–12.63)	50.52 (48.13–52.91)	1.45 (1.04–1.86)	1.21 (0.84–1.58)	4.79 (4.05–5.53)
6	2.19 (1.69–2.69)	7.76 (6.83–8.69)	34.9 (32.92–36.88)	0.7 (0.42–0.98)	0.73 (0.44–1.02)	2.74 (2.19–3.29)
7	1.6 (1.18–2.02)	6.01 (5.19–6.83)	23.88 (22.25–25.51)	0.7 (0.42–0.98)	0.52 (0.28–0.76)	2.5 (1.97–3.03)
8	1.54 (1.13–1.95)	3.74 (3.09–4.39)	17.18 (15.8–18.56)	0.46 (0.23–0.69)	0.35 (0.15–0.55)	1.63 (1.2–2.06)
9	1.23 (0.86–1.6)	3.62 (2.98–4.26)	13.69 (12.45–14.93)	0.29 (0.11–0.47)	0.35 (0.15–0.55)	1.66 (1.23–2.09)
10	1.33 (0.94–1.72)	2.28 (1.77–2.79)	10.64 (9.54–11.74)	0.27 (0.09–0.45)	0.33 (0.14–0.52)	1.16 (0.8–1.52)
11	0.99 (0.65–1.33)	2.35 (1.83–2.87)	8.26 (7.28–9.24)	0.36 (0.16–0.56)	0.3 (0.11–0.49)	1.24 (0.86–1.62)
12	0.49 (0.25–0.73)	2.02 (1.53–2.51)	6.27 (5.41–7.13)	0.24 (0.07–0.41)	0.18 (0.03–0.33)	1.04 (0.69–1.39)
13	0.75 (0.45–1.05)	1.71 (1.26–2.16)	5.1 (4.32–5.88)	0.22 (0.06–0.38)	0.09 (–0.01–0.19)	0.9 (0.57–1.23)
14	0.63 (0.35–0.91)	1.58 (1.14–2.02)	5.25 (4.45–6.05)	0.09 (− 0.01–0.19)	0.09 (− 0.01–0.19)	0.51 (0.26–0.76)

^a^Annual hospitalization rates expressed per 100,000 population (95% CI)

**Fig. 3:**
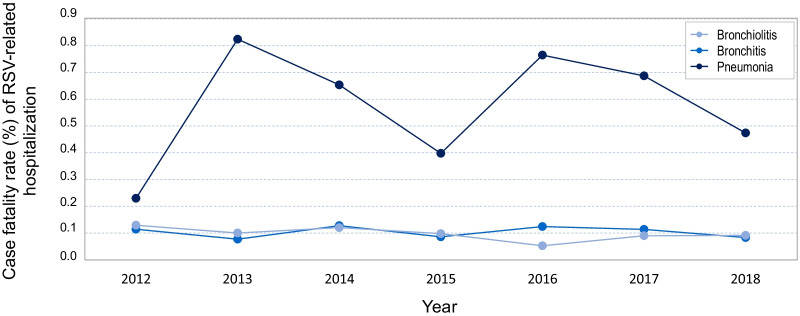
Trend over time of case fatality rate (%) of RSV-associated bronchiolitis, bronchitis, and pneumonia in children up to 14 years of age in Spain from 2012 to 2018

The case fatality rate among hospitalized children diagnosed with pneumonia due to RSV was almost six times higher than among those diagnosed with bronchiolitis or bronchitis throughout the study period, 0.57% vs. 0.1% (for both diagnoses). Unlike the case fatality rates in children with bronchiolitis and bronchitis, that were comparable throughout the 7-year study period, the case fatality rate in children with pneumonia was more variable, showing maximum values in the years 2013 and 2016 (Fig. 3).

## Discussion

In this population-based study we provided data on the burden of hospitalizations for RSV-related diseases in children up to 14 years of age in Spain from 2012 to 2018. Our results show that RSV-related diseases represent a heavy burden for both the patients and the health care system, and highlight the urgent need to implement diagnostic and therapeutic interventions to prevent RSV infection in children, especially among those at higher risk. Almost 200,000 children were admitted in Spain for respiratory disease, including RSV infection, bronchiolitis, bronchitis and pneumonia, mainly children younger than one year of age. The main cause of hospitalization was bronchiolitis, 62.4% of all diagnoses, of which more than 60% were associated with RSV infection. We estimated a mean annual rate of 221.0 bronchiolitis hospitalizations per 100,000 children among the general population, and 140.7 per 100,000 children when only RSV-associated bronchiolitis was considered. By age group, these rates were highest among children under 12 months of age—the most vulnerable population according to published evidence [3, 11]—, reaching 1296 hospitalizations per 100,000 children for ‘acute bronchiolitis’ and 2185 for ‘bronchiolitis due to RSV’. Although almost a third of all recorded hospitalizations were for bronchitis, only 1.6% were associated with RSV infection, resulting in a mean annual rate of only 5.7 hospitalizations per 100,000 children. A similarly low percentage of cases (1.9%) and low hospitalization rate (7.2/100,000) were estimated for RSV-associated pneumonia. Young age is a known risk factor for RSV infection, with about 45% of hospital admissions and in-hospital deaths due to RSV-related bronchiolitis occurring in children younger than 6 months [17, 35]. Previous studies have shown that the highest incidence of childhood hospitalization for bronchiolitis in Spain in the last 20 years occurred during the first year of life, particularly among children aged 0–2 months, estimated at about 4000 cases per 100,000 children under 12 months of age; rates decrease significantly thereafter [19, 36]. Estimates published for different countries around the world are variable, probably due to different healthcare settings and estimation methods, and to the different underlying characteristics of the study populations [21, 37–43]. However, it is interesting to note that with all of these approaches and in all settings, the highest rates were recorded in the youngest age group, an important consideration for targeting interventions.

In this study, we observed notable differences in the mean annual incidence of hospitalization between RSV-associated bronchiolitis, bronchitis, and pneumonia that generally remained constant throughout the study period. Bronchitis usually affects older children and adults, while bronchiolitis is more common in younger children, most often under 2 years of age. However, the potential existence of an age-associated bias in the diagnosis of children admitted for RSV-associated respiratory disease has been suggested elsewhere. According to these authors, RSV-associated hospital admissions may be more likely to be coded as bronchiolitis in children younger than 6 months, but as pneumonia or unspecified ALRI in children older than 1 year [44]. On the other hand, a diagnosis of RSV-associated pneumonia is very unusual. Laboratory tests are not always performed to confirm the diagnosis or are delayed until clinicians consider that the recovery of hospitalized children is too prolonged [45, 46].

The issue of pediatric hospital discharges for respiratory diseases to which a generic diagnostic code is assigned, e.g., bronchitis or bronchiolitis, has been addressed previously. In a recently published study [36], we collected data from all patients who had been assigned a generic diagnostic code for bronchiolitis, assuming that 50–80% of cases were caused by RSV infection. In this study, however, the patients were analyzed in different groups based on whether the diagnosis code was generic or specifically RSV-positive. We found that between 61% (2012) and 73% (2016) of hospital admissions for bronchiolitis had been classified as RSV-positive, but only between 1% (2018) and 7% (2015) of hospital admissions for bronchitis were classified as RSV-positive, instead of the expected 50–80% [1, 47], showing that, very often, RSV infections are inappropriately coded in children hospitalized for bronchitis.

Using joinpoint regression analysis, no evidence of statistically significant changes in the mean annual hospitalization rate for bronchiolitis or bronchitis, or changes in the trend, was found throughout the study period. However, we found a linear increase in the mean hospitalization rate for pneumonia of almost 15% per year from 2012 to 2018. Perhaps improvements in diagnostic techniques in recent years, the increased survival of very preterm infants in the perinatal period, environmental changes, or even subtle changes in the organization of healthcare prompting parents to seek hospital care have contributed to this increasing trend in the rate of hospitalizations for pneumonia [48–50]. Based on the stable pediatric intensive care admission rates reported in some studies [50], some authors suggest that it seems unlikely that the growing trend is due to an increase in the severity of infection or the virulence of RSV in children. Other authors suggest that this increase might reflect changes in the threshold for admission (particularly in the younger infants) or failure to manage these acute illnesses in the community care setting [44]. In any case, more research is needed to shed light on this issue.

The mean case fatality rate in our study was almost six times higher in children hospitalized with a diagnosis of RSV-positive pneumonia (mean proportion, 0.57%) than in children hospitalized with a diagnosis of bronchitis or bronchiolitis (mean proportion, 0.10% for the two diagnoses). The difference in hospitalization rates between diagnoses was quite variable throughout the study period and, with the exception of 2012, highly relevant. However, we believe there could be a patient selection bias, as previously mentioned. Although viruses are the most common cause of pediatric pneumonia—especially in infants younger than 2 years—, microbiological testing is only performed if the child’s progress during their hospital stay is slow, usually related to the presence of known risk factors or any other pediatric alert that identifies hospitalised children at risk of clinical deterioration. Thus, other patients with RSV-positive pneumonia could be lost in the diagnostic selection due to lack of laboratory tests. Most studies do not report detailed cause of death and clinical details of the fatal cases are often unavailable, limiting our ability to accurately assess RSV-related infant mortality [35, 51].

We also found statistically significant sex differences in the rate of hospital admissions for bronchiolitis and bronchitis (1.3 times higher in males than in females for both diagnoses, *p* < 0.001), but not for cases of viral pneumonia. Male sex is a known risk factor for the development of respiratory tract infections, and generally presents with a more severe course than in females, leading to higher mortality rates. It has been reported that male infants have shorter and narrower airways and therefore are more likely to develop bronchial obstruction after contracting RSV infection [52]. Pediatricians must take these sex differences into account when treating children with RSV respiratory infections [21, 53].

This study has several strengths and limitations that should be considered. Its main strength lies in the use of MBDS records that offer a very large sample size that includes practically all admissions to both public and private acute hospitals in Spain, thus increasing the power of the statistical analyses, even for low-prevalence diseases. Furthermore, the methodology used has been well recognized for many years and offers great external validity, making the MBDS a valuable tool for epidemiological research into respiratory diseases [54]. On the other hand, one of the main limitations when interpreting the MBDS data is that its reliability depends on the quality of the discharge report and the clinical history and on the codification process variables [55]. Some of the ICD codes used, such as 466.1 (acute bronchiolitis), do not restrict the search to RSV-positive cases only, which means that children hospitalized for respiratory diseases caused by other respiratory pathogens have probably been included in this study. Many children are discharged before the results of laboratory tests for RSV infection are available, or they are simply coded as ‘acute bronchiolitis’ even though they are positive for RSV. Therefore, the use of more restrictive codes to gather only confirmed RSV-positive cases would have meant the loss of a significant part of the RSV-related disease burden [56]. Another potential limitation of this study is that it was restricted to hospitalized patients. As previously described, the majority of recurrent wheezing and RSV-related burden of disease occur in the outpatient setting [3], so our results may have underestimated, at least partially, the true impact of RSV-related respiratory disease in the population.

In summary, RSV-related diseases represent a heavy burden to children, their families, and the public health system in Spain. To address this problem, there is consensus that early protective measures should be applied, especially in children during their first 6–12 months of life. These measures include general hygienic practices, such as visiting restrictions during the RSV outbreak period and effective handwashing, and, where available, immunotherapy for all children, since it has been shown that the majority of infected children were full-term and otherwise healthy [2, 57, 58]. With the introduction of RSV vaccine, documenting how such vaccines impact the incidence of RSV-related diseases will help clarify the relationship between RSV infection and subsequent recurrent wheezing, particularly in the long-term [56].

## Conclusions

- RSV-related respiratory infections remain a leading cause of infant hospitalization in Spain.
- Effective antiviral treatments and preventive vaccines are urgently needed for the management of RSV infection in children, especially for those aged 6–12 months.

## Data Availability

The datasets generated and/or analysed during the current study are available in the Hospital Discharge Records in the Spanish National Health System (CMBD) repository, available at: https://www.mscbs.gob.es/en/estadEstudios/estadisticas/cmbdhome.htm. The information contained in this repository is in the public domain and can be accessed without the need for any administrative permissions.

## Abbreviations

AAPC: Average annual percentage change
ALRI: Acute lower respiratory tract infection
ANOVA: Analysis of variance
APC: Annual percentage change
CI: Confidence interval
FDA: US Food and Drug Administration
ICD: International classification of diseases
ICD‑9‑CM: International classification of diseases 9th Revision, Clinical Modification
ICD‑10‑CM: International classification of diseases 10th Revision, Clinical Modification
ICU: Intensive care unit
IgG: Immunoglobulin G
IP: Immunoprophylaxis
IQR: Interquartile range
MBDS: Minimum Basic Data Set
RSV: Respiratory syncytial virus
